# Relationships between Regional Radiation Doses and Cognitive Decline in Children Treated with Cranio-Spinal Irradiation for Posterior Fossa Tumors

**DOI:** 10.3389/fonc.2017.00166

**Published:** 2017-08-18

**Authors:** Elodie Doger de Speville, Charlotte Robert, Martin Perez-Guevara, Antoine Grigis, Stephanie Bolle, Clemence Pinaud, Christelle Dufour, Anne Beaudré, Virginie Kieffer, Audrey Longaud, Jacques Grill, Dominique Valteau-Couanet, Eric Deutsch, Dimitri Lefkopoulos, Catherine Chiron, Lucie Hertz-Pannier, Marion Noulhiane

**Affiliations:** ^1^INSERM U1129, CEA, Paris Descartes University, Paris, France; ^2^UNIACT, Institut Joliot, DRF, Neurospin, CEA, Paris Saclay University, Gif-sur-Yvette, France; ^3^Department of Pediatric and Adolescent Oncology, Gustave Roussy, Villejuif, France; ^4^Radiation Oncology Department, Gustave Roussy Cancer Campus, Villejuif, France; ^5^INSERM, U1030, Villejuif, France; ^6^Paris Sud University, Paris-Saclay University, Villejuif, France; ^7^Gustave Roussy, Paris-Saclay University, Department of Medical Physics, Villejuif, France; ^8^INSERM U992 Unicog CEA, Neurospin, Paris Descartes University, Paris, France; ^9^Institut Joliot, Neurospin, CEA, Paris-Saclay University, Gif-sur-Yvette, France; ^10^CSI Department for Children with Acquired Brain Injury, Hopitaux de Saint Maurice, Saint-Maurice, France; ^11^Paris Sud University, Orsay, France

**Keywords:** pediatric, posterior fossa, radiotherapy, cognitive impairments, radiation effects

## Abstract

Pediatric posterior fossa tumor (PFT) survivors who have been treated with cranial radiation therapy often suffer from cognitive impairments that might relate to IQ decline. Radiotherapy (RT) distinctly affects brain regions involved in different cognitive functions. However, the relative contribution of regional irradiation to the different cognitive impairments still remains unclear. We investigated the relationships between the changes in different cognitive scores and radiation dose distribution in 30 children treated for a PFT. Our exploratory analysis was based on a principal component analysis (PCA) and an ordinary least square regression approach. The use of a PCA was an innovative way to cluster correlated irradiated regions due to similar radiation therapy protocols across patients. Our results suggest an association between working memory decline and a high dose (equivalent uniform dose, EUD) delivered to the orbitofrontal regions, whereas the decline of processing speed seemed more related to EUD in the temporal lobes and posterior fossa. To identify regional effects of RT on cognitive functions may help to propose a rehabilitation program adapted to the risk of cognitive impairment.

## Introduction

Posterior fossa tumors (PFTs) account for two-thirds of all pediatric brain tumors (1). The most common malignant PFT is medulloblastoma (40%), followed by ependymoma (10%) (2). As a result of improved treatment, event-free survival has significantly increased (3). However, these children suffer from varied cognitive impairments, the most frequently described being decreased sustained attention, working memory, and information processing speed (4). This latter impairment seems to appear first in PFT patients treated with cranio-spinal irradiation (CSI) (4). These cognitive impairments might relate to the decline of global intellectual functioning (full scale IQ, FSIQ) reported to be between two and four points per year (5–9). Several factors have been shown to predict cognitive impairments in PFT patients. Radiotherapy (RT) has been considered to be the major one, especially in young children (6, 8). Three RT-associated risk factors have been highlighted as predictors of cognitive impairments: (i) CSI (6, 7, 10), (ii) the volume receiving the boost [i.e., to the posterior fossa (PF)] (11), and (iii) the dose per fraction (12, 13). Grill et al. (10) observed that PFT survivors with low CSI (25 Gy) showed better cognitive outcomes than those receiving high CSI (36 Gy). Nonetheless, the reduction of CSI dose (14) did not prevent IQ decline (9). An alternative way to decrease cognitive impairments has been to reduce the volume of the PF irradiated, in addition to the reduced CSI. While the PF received the highest dose, the boost dose also contributed to higher doses in other regions such as the temporal lobes, the brainstem, and the hypothalamus (11). Moxon-Emre et al. (15) showed that medulloblastoma survivors for whom the CSI was reduced, and the boost volume was reduced from the entire PF to the tumor bed, had preserved IQ over time. Nonetheless, medulloblastoma survivors treated *via* either a CSI dose reduction or a diminution of PF volume irradiated (tumor bed boost) still experienced a decline of IQ.

Recent studies reported a higher contribution of specific brain regions to the development of RT-induced cognitive decline. Jalali et al. (16) observed that more than 43.2 Gy to >13% of the left temporal lobe was predicting IQ decline in patient treated for a benign tumor with stereotactic conformal RT. Merchant et al. (6) assessed the impact on IQ change of different mean dose values in distinct regions (whole brain, temporal lobe, hippocampus, infratentorial, and supratentorial spaces) in patients treated for a medulloblastoma, and suggested that supratentorial space was the most sensitive across the brain. Using a neurocognitive questionnaire, Armstrong et al. (17) pointed out a strong association between maximum radiation dose to the temporal lobe and poor performance in *Task efficiency* (i.e., attention and processing speed) and *Organization*. These subscores were measured as given by the Childhood Cancer Survivor Study Neurocognitive Questionnaire. While these studies did not identify a relationship between radiation dose of PF and changes in cognitive scores, such associations have been reported in children with ependymoma (18).

Despite marked progress, the regional effect of RT on cognitive impairment still remains unclear. So far, research on this question has been mainly carried out on either single (19) or large (6) brain regions, limiting the analysis to specific anatomical structures. In this study, we implemented a whole brain analysis; to investigate the relation between regional biological dose and changes over time of different cognitive scores (IQ, processing speed, and working memory) in 30 patients treated for a PFT. The use of a principal component analysis (PCA) was an innovative way to cluster correlated irradiated regions due to similar radiation therapy protocols across patients. We aimed to describe the relationships between regional radiation dose and declines in specific cognitive functions.

## Patients and Methods

### Patient’s Characteristics

Inclusion criteria were (1) PFT patients treated at Gustave Roussy Cancer Campus between 2000 and 2014; (2) 17 years of age or under at diagnosis (3) multiple (>2) IQ assessments after treatment onset (4); for the PFT patients treated with radiation therapy, the computed tomography (CT) scan, T1-weighted MRI, and dosimetric maps had to be available. Thirty patients (14 males and 16 females) matched these criteria. Information was gathered from medical files about the history of the illness (i.e., age at diagnosis) and the type of treatment (i.e., surgery, chemotherapy, and radiation therapy protocol). The underlying malignancy of the 30 patients studied was medulloblastoma, ependymoma, astrocytoma, and embryonal tumor in 25, 3, 1, and 1 patients, respectively. Twenty patients had a localized disease and 10 had a metastatic disease. Complete tumor resection was achieved in 20 PFT. Post-operative complications occurred in 10 patients. No patient relapsed between the two evaluations, but the patient with an astrocytoma whose relapse before the first evaluation, was treated with chemotherapy alone. The mean age at diagnosis was 4.62 years (SD = 3.05; [0.49; 12.24]). The mean delay between treatment and the last assessment was 4.60 years (SD = 4.60; [1.28; 14.24]). Pre-operative hydrocephalus was present in 19 patients (63%). Seventeen patients were treated with RT alone (*N* = 7) or RT and chemotherapy (*N* = 10). The remaining patients were treated with chemotherapy alone and were used as controls. All patients with RT received a CSI and a boost in the PF and were treated with three-dimensional conformal radiation therapy (Table 1). This study was approved by an ethical committee (CPP no. 14973, Gustave Roussy, Villejuif, France).

**Table 1:** Absorbed dose and type of fractionation [conformational fractionation (CF) vs. hyperfractionated radiotherapy (HFRT)] prescribed to the cranio-spinal irradiation (CSI) and posterior fossa (PF) for the 17 patients.

Patients	CSI (Gy)	PF (Gy)	Fractionation
Patient 1	18	54	CF
Patient 2	18	54	CF
Patient 3	25.2	50.4	CF
Patient 4	18	36	CF
Patient 5	18	55.4	CF
Patient 6	18	50.4	CF
Patient 7	36	54	CF
Patient 8	25.2	54	CF
Patient 9	36	68	HFRT
Patient 10	36	68	HFRT
Patient 11	18	45	CF
Patient 12	36	54	CF
Patient 13	36	68	HFRT
Patient 14	36	68	HFRT
Patient 15	36	68	HFRT
Patient 16	36	68	HFRT
Patient 17	36	68	HFRT

*The number of fractions per day and the dose per fraction varied from one patient to another. Some patients received two fractions of 1 Gy per day with an inter fraction of 8 h with HFRT, whereas other patients were treated by CF, i.e., one fraction of 1.82 Gy per day*.

### Neuropsychological Assessments

Three cognitive indices were estimated from age appropriate Wechsler Intelligence Scale (20, 21): FSIQ in all patients and the processing speed index (PSI) and working memory index (WMI) when available. Neuropsychological assessments were done by formally trained neuropsychologists from the pediatric department. Because of age or time constraints, not all participants were administered all the tests. Thus, the numbers of patients assessed varied across cognitive scores [*N* (ΔFSIQ) = 30, *N* (ΔPSI) = 23, *N* (ΔWMI) = 14]. Patients were evaluated at variable time points after treatment onset. Thus, the delay (T) between two neuropsychological assessments varied from one patient to the other (Table 2). The change in cognitive scores (ΔFSIQ, ΔPSI, and ΔWMI) of each patient was calculated from the difference between the first and last scores (ΔT). We did not consider intermediate scores. Changes in cognitive scores (ΔFSIQ, ΔWMI, and ΔPSI) were compared using two-tailed Student’s *t*-tests.

**Table 2:** Changes in the three measured cognitive scores [Delta (Δ)] with the corresponding number of evaluated patients: mean score change (±SD, range) and mean test interval ΔT (±SD, range).

Δ scores	*N*	Mean (±SD)	Range	Mean [ΔT in years (±SD)]	Range
ΔFIQ	30	−2.03 (11.70)	[−29; 28]	3.97 (±2.74)	[1.00; 12.29]
ΔPSI	23	−0.6 (14.44)	[−28; 41]	3.74 (±2.30)	[0.89; 9.93]
ΔWMI	14	−3.66 (9.15)	[−24; 6]	2.81 (±1.85)	[1.00; 8.14]

## Neuroimaging Data

To study regional dose effects on changes in cognitive scores, 3D-T1 MRI, CT scan, and absorbed dose maps of patients treated with RT (*n* = 17) were collected and processed to create individual dose distribution maps into selected brain regions of interest (ROI) covering the whole brain.

### Image Collection

3D T1-MR images were acquired on a 3-T scanner using a 32-channel head coil (General Electric, Milwankee, MN, USA). In this clinical retrospective study, two types of T1-weighted images were collected: 3D T1-weighted sagittal slices (matrices: 256 mm × 256 mm, pixel size: 0.5 mm, slice thickness: 1 mm, FOV: 240 mm) and 3D-T1 weighted axial slices (matrices: 224 × 288, pixel size: 0.5 mm, slice thickness: 1 mm).

Computed tomography scans were acquired on a SIEMENS Sensation Open scanner located in Gustave Roussy RT department (matrices: 512 mm × 512 mm, pixel size: 0.8 mm, slice thickness: 3 mm). Radiation dose maps (RD maps) were computed with the ISOgray™ Treatment Planning System (DOSIsoft, version 4.1, Cachan, France). The Clarkson–Cunningham model was used for dose calculation. Dose maps resolution was 3 mm × 3 mm × 1 mm.

### Image Analysis

#### Image Preprocessing

**Figure 1:**
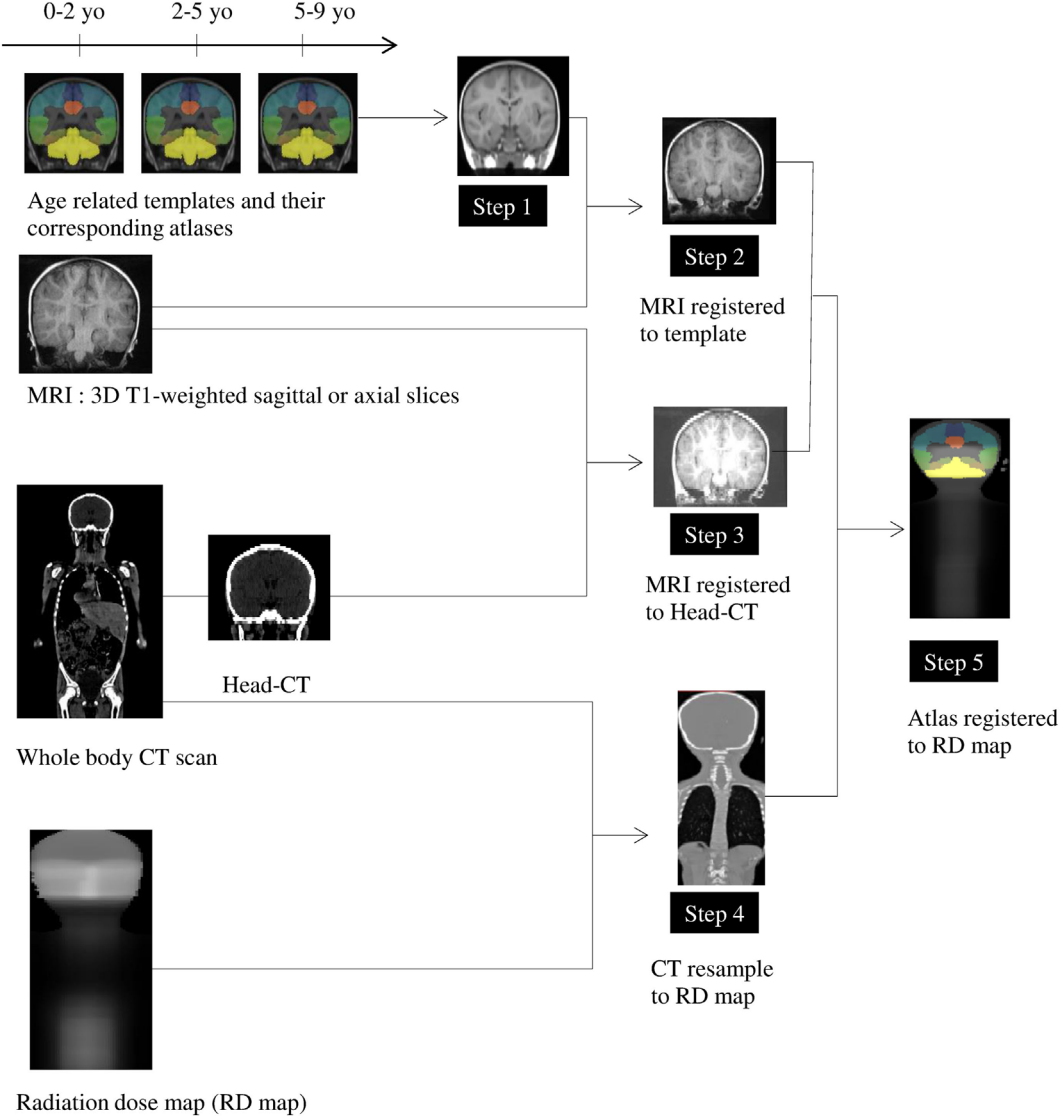
Preprocessing pipeline (see Patients and Methods). *Step 1*: Selection of age appropriate templates. *Step 2*: Registration of the selected template on individual patient 3DT1 image. *Step 3*: Registration of individual 3DT1 image to the corresponding individual CT. *Step 4*: Down-sampling of CT to match the corresponding radiation dose map. *Step 5*: Registration of the selected template on the individual dose map coordinate system.

We designed a five-step preprocessing pipeline to identify anatomical ROI on dose maps (Figure 1).

*Step 1*: We chose three MRI templates specific to ages 0–2, 2–5, and 5–9 years (151 × 192 × 152 voxels, 1 mm × 1 mm × 1 mm voxel size) and three corresponding anatomical atlases (151 × 192 × 152 voxels, 1 mm × 1 mm × 1 mm) from the Neurodevelopmental MRI DataBase (22). The atlases contained 56 ROIs extracted from the LPBA40 atlas (23) that were adapted to selected ages thanks to label propagation and decision fusion methods (24). For each child, we selected both the atlas and associated template according to the age at which the child received radiation therapy, to be as close as possible to the individual anatomy, which varies significantly during development (22, 25). Since the atlases did not included some particular regions (i.e., corpus callosum, a part of internal capsule, and ventricles), we created a supplementary label that encompassed these regions, resulting in 57 ROIs.

*Step 2*: The selected template was warped to individual patient 3DT1 image using a non-linear registration tool [Advanced Normalization Tools, SyN (26), and ANTS (27)].

*Step 3*: Individual MR images were also registered to the corresponding individual CT scan by applying a linear transformation with FSL [FLIRT (28)].

*Step 4*: Each CT scan was then down sampled to match the corresponding RD map voxel sampling.

*Step 5*: Finally, we combined the computed transformations into a single concatenated transformation from the template space to the individual dose map coordinate system. This enabled us to perform statistical radiation dose analyses over the group in each ROI extracted from the template.

Individual registrations have been assessed qualitatively by two experimenters independently and by consensus. From this check, four subjects were excluded from the study. In the majority of cases, registrations have been adjusted manually to optimize intersubject comparisons.

#### Data Analysis

**Figure 2:**
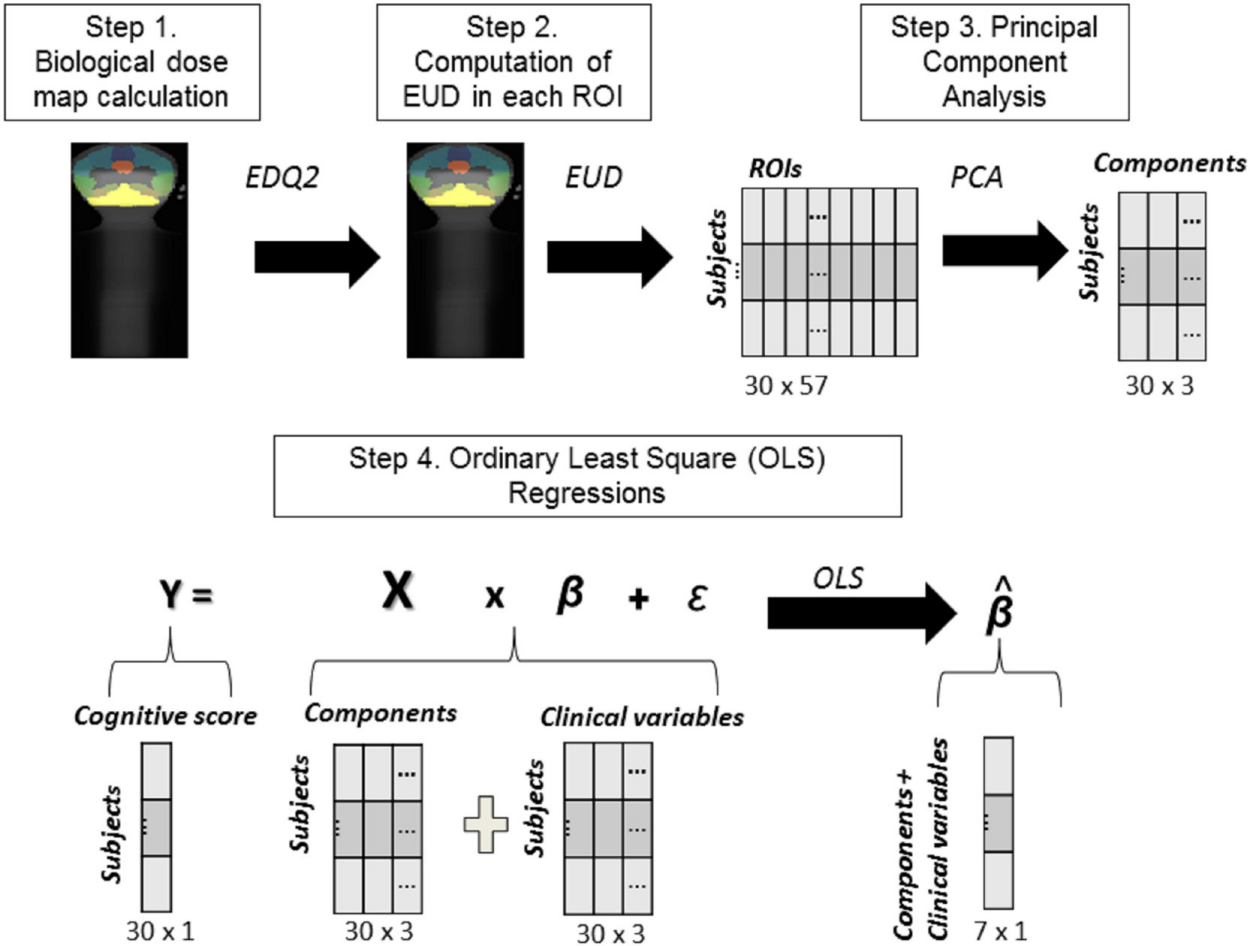
Steps of the analysis. *Step 1*: Equating dose maps across patients: EQD_2_ computation. *Step 2*: Calculation of dose index in each ROI: equivalent uniform dose computation. *Step 3*: Principal component analysis approach. *Step 4*: Highlighting the respective contribution of clinical variables and PC-_EUD_ on clinical scores changes using ordinary least square regression.

We designed a four-step analysis pipeline to determine the associations between both clinical variables and ROI dose distribution with changes in cognitive scores (Figure 2).

##### Equating Dose Maps across Patients: EQD_2_ Computation

*Step 1*: Given the differences in fractionation parameters (dose per fraction and number of fractions per day varied from one patient to another), even at equal total doses, the biological effectiveness of these two types of irradiation will be different (Figure 2, step 1). However, using the linear quadratic model (29), it is possible to calculate the total dose equivalent in terms of biological effects for two different fractionations (dose per fraction, time interval between two fractions) and a given tissue (EDQ2). Using this equation, all treatments are thus reduced to biological dose equivalent to treatments performed with fractions of 2 Gy, which is the standard fractionation scheme. Therefore, we corrected the dose of all fractionation types in a uniform way by calculating in each voxel the equivalent dose with the EQD_2_ formula (30) (Eq. 1; Figure 2, step 1). The EQD_2_ was calculated taking into account a function (*H_m_*) depending on the number of equally spaced fractions per day; the dose per fraction (*d*) and the sensitivity of the tissue (α/β). *D* (the total delivered dose in Gy) and *d* varied across patients. Based on the current literature, α/β was fixed to 2 and T1/2 to 3 h (31):

(1) $$\text{EQD}2=D.\frac{d(1+H_{m})+\alpha /\beta }{2+\alpha /\beta }$$

##### Calculation of Dose Index: Equivalent Uniform Dose (EUD) Computation

*Step 2*: After calculating the biological dose map of each patient, for all subjects and ROIs we computed the EUD that accounts for heterogeneity of dose distribution, as follows (Eq. 2) (Figure 2, step 2):

(2) $$\text{EUD}={\left(\sum_{j}v_{j}D_{j}^{k}\right)}^{\frac{1}{k}}.$$

Equivalent uniform dose corresponds to the value of a homogeneous dose that would cause the same clinical effect than the corresponding heterogeneous dose distribution (30). *k* was fixed at 5 according to the work of Emami et al. (32). We standardized EUD across the 17 subjects for each of the 57 ROIs.

##### Taking into Account the Spatial Correlation of Radiation Doses across ROIs: PCA Approach

*Step 3*: Because of the radiation therapy protocol (i.e., CSI and boost in the PF with three-dimensional conformal radiation therapy), EUD was highly correlated across brain regions (Figure 2, step 3). Therefore, it was not possible to assess the effect of irradiation on cognitive scores in each region with an ordinary least square regression, as regression weights would be highly unstable. Thus, we ran a PCA, a data-driven method that clusters correlated variables into common factors named principal components (PCs). In this approach, highly correlated variables share higher weights within each factor/component, but components are uncorrelated. The PCA enabled us (1) to obtain uncorrelated components representative of the radiation dose distribution variability across subjects, each component revealing a brain network with a particular radiation pattern and (2) to reduce the number of variables in our model, as sample size was limited. We performed a PCA taking the ROIs normalized EUD as variables (Figure 2, step 3). Then, we selected the *n* < 57 PCs accounting for 90% of the variance (33). Due to the high correlation between regions, we recovered only three components (PCs). To figure out the spatial contribution of the ROIs on each PC-_EUD_, we computed the correlations between EUD in each ROI and each PC-_EUD_, and projected the correlation coefficients onto a glass brain.

#### Highlighting the Respective Contribution of Clinical Variables and EUD-PCs on Clinical Score Changes

*Step 4*: We then considered the computed PCs-_EUD_ and the clinical variables (chemotherapy, time since diagnosis, age at diagnosis, and ΔT) in a least square regression (Figure 2, step 4). We first checked for multicollinearity that could induce a biased estimation and a loss of power (34), using the variance inflation factor, which summarizes how an independent variable is explained by other variables. We removed regressors with a variance inflation factor >10 (35). In each regression, we examined *t*-scores to determine which variable had the most important effect on the cognitive scores of these 30 patients.

All analyses and plots were computed using the *Python libraries, Nilearn, Scikit Learn*, and *Statsmodels* (36, 37).

## Results

### Neuropsychological Performance

At time of first neuropsychological assessment, the mean estimated IQ over the whole population was 87.5 (SD = 18.4; [45–130]). A declining performance over time was observed in 67, 64, and 48% of the patients for ΔFSIQ, ΔWMI, and ΔPSI, respectively. The remaining patients showed either preserved or better performance over time. However, there were no statistically significant differences between cognitive scores [ΔFSIQ vs. ΔWMI: *t*(32) = −0.64, *p* = 0.52; ΔPSI vs. ΔWMI: *t*(35) = −0.81, *p* = 0.42; ΔFSIQ vs. ΔPSI: *t*(51) = −0.37, *p* = 0.70] (Table 2). Moreover, ANOVAs were conducted to compare the three treatment groups (chemotherapy alone vs. RT and chemotherapy vs. RT alone) on their cognitive scores (ΔFSIQ, ΔWMI, and ΔPSI). There was no statistically significant difference between treatment groups in ΔFSIQ [*F*(2,30) = 2.36; *p* = 0.11; RT alone: M = −10.5 (±9.11), chemotherapy alone: M = 1.57 (±11.28), RT and chemotherapy: M = −2.0 (±11.03)]; and ΔWMI [*F*(2,14) = 1.17; *p* = 0.34; RT alone: M = −10 (±9.90), chemotherapy alone: M = −0.29 (±5.90), RT and chemotherapy: M = −4.60 (±10.15)], and ΔPSI [*F*(2,24) = 2.28; *p* = 0.12; RT alone: M = −10 (±9.90), chemotherapy alone: M = −0.29 (±5.90), RT and chemotherapy: M = −4.60 (±10.15)].

### PCs Extracted from EUD of Anatomical ROIs

*PC1-_EUD_*, which explained 67% of the variance of original data, was strongly correlated (>0.50) with the dose (EUD) in all regions, especially in the supratentorial space. *PC2-_EUD_* explained 19% of the variance and was positively correlated with 16 regions in the PF, inferior occipital and temporal regions (see Table S1 in Supplementary Material). Meanwhile, three regions in the left superior occipital and parietal regions correlated negatively and moderately (>0.40) with *PC2-_EUD_* (see Table S1 in Supplementary Material). *PC3-_EUD_* explained 5% of the variance and had a moderate positive correlation (>0.40) with the EUD in the left orbitofrontal area. By contrast, the precuneus and the right cuneus negatively correlated with *PC3-_EUD_*. Values of all correlation coefficients are shown in Table S2 in Supplementary Material.

### Effects of Clinical Variables and EUD Components on Cognitive Score Changes

Our final regression models included the three *PCs-_EUD_*, chemotherapy, age at diagnosis, and delay between assessments (ΔT). Indeed, in all models, time since diagnosis (variance inflation factor >10) was highly correlated with ΔT (variance inflation factor >10), while it was not the case between PCs (variance inflation factor <10), chemotherapy (variance inflation factor <10), and age at diagnosis (variance inflation factor <10). We thus removed time since diagnosis from the analysis and checked that all remaining variance inflation factor indices were below 10.

#### Clinical Variables and Cognitive Score Changes

ΔFSIQ was significantly negatively affected by age at diagnosis and interval between assessments (ΔT), and positively influenced by chemotherapy. These variables had no significant effect on the other scores (Table 3).

**Table 3:** Effects on changes of cognitive scores (ΔFSIQ, ΔPSI, and ΔWMI) of the clinical variables and the components of the principal component analysis, according to our models (see Patients and Methods).

Δ score	*N*	Age at diagnosis	ΔT	Chemotherapy	*EUD_PC1_*	*EUD_PC2_*	*EUD_PC3_*	*R*^2^
ΔFSIQ	30	−2.14 (0.04)	−2.26 (0.04)	3.08 (0.01)	−0.35 (0.73)	−1.98 (0.06)	−1.98 (0.06)	0.43
ΔPSI	23	−1.38 (0.18)	−0.31 (0.76)	1.49 (0.15)	−1.15 (0.27)	−2.31 (0.03)	−2.05 (0.05)	0.43
ΔWMI	14	0.44 (0.67)	−1.32 (0.22)	0.44 (0.67)	−3.13 (0.01)	−2.12 (0.06)	−4.09 (0.0001)	0.80

*For each variable, *t* score and *p* value (under parenthesis) are given, and for each model, the adjusted *R*^2^ indicates the total proportion of the scores variance that was predicted from the variables*.

#### EUD Components and Cognitive Score Changes

**Figure 3:**
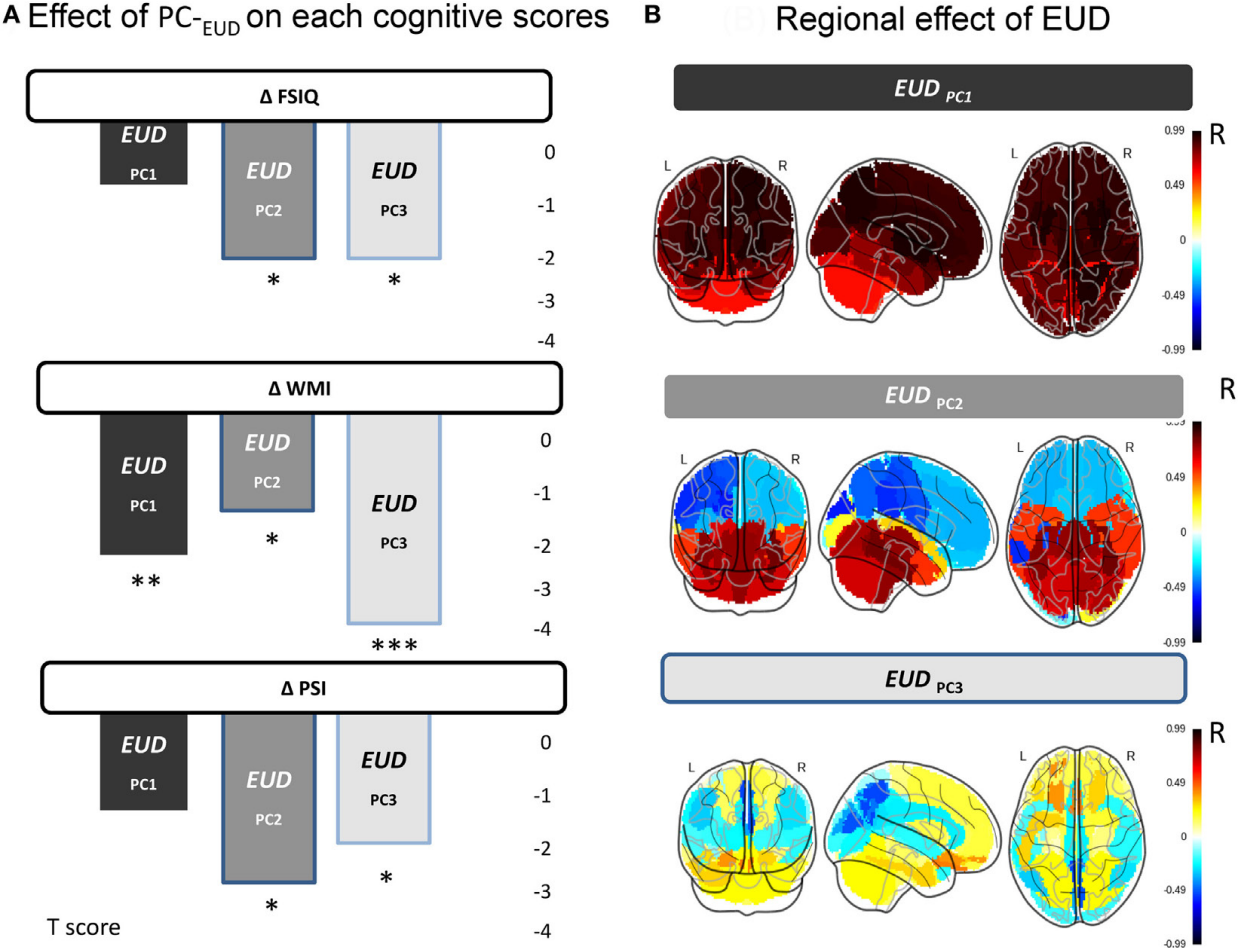
Summary of results: impact of equivalent uniform dose (EUD) principal components on cognitive changes (ΔFSIQ, ΔPSI, and ΔWMI). **(A)** Effects of EUD components on each cognitive score (ΔFSIQ, ΔPSI, and ΔWMI). The weights of each PC-_EUD_ on the cognitive change are displayed in gray color scale, with significance levels (*p ≤ 0.05: **p ≤ 0.01: ***p ≤ 0.001). **(B)** Regional effects of EUD. The color scale displays the regional correlation coefficients *R* between EUD and the PC-_EUD_ in each ROI, i.e., the relative participation of each ROI on each EUD component (with higher positive correlations shown in red, stronger negative correlations in blue).

ΔWMI was clearly negatively associated with both *PC1-_EUD_* and *PC3-_EUD_* and marginally by *PC2-_EUD_* (Table 3) *PC3-_EUD_* had the highest effect on ΔWMI, followed by *PC1-_EUD_* and *PC2-_EUD_* (Figure 3). The decline of WMI was first associated with an increase of EUD in left orbitofrontal area (*PC3-_EUD_*) and then with an increase of EUD in all regions, especially in the supratentorial space (*PC1-_EUD_*). By contrast, an EUD increase in the precuneus and right cuneus was positively associated with ΔWMI (Figure 3).

Only *PC2-_EUD_* and *PC3-_EUD_* were found to have a negative and significant effect on ΔPSI, with a seemingly higher effect of *PC2-_EUD_* than of *PC3-_EUD_*, contrarily to ΔWMI (Table 3). The decline of PSI was first associated with an EUD increase in the PF, inferior occipital and temporal regions (*PC2-_EUD_*) followed by an increase in the left orbitofrontal area (*PC3-_EUD_*). By contrast, *PC2-_EUD_* and *PC3-_EUD_* were positively associated with ΔPSI in superior occipital and parietal regions (Figure 3).

Finally, *PC2-_EUD_* and *PC3-_EUD_* had similar and nearly significant negative effects on ΔFSIQ (Table 3) The decline of FSIQ was similarly associated with the increase of EUD in the PF, inferior occipital, temporal regions, and left orbitofrontal areas (*PC3-_EUD_* and *PC2-_EUD_*). By contrast, EUD in superior occipital and parietal regions was positively associated with ΔFSIQ (*PC2-_EUD_* and *PC3-_EUD_*) (Figure 3).

## Discussion

Our main results suggest different regional associations between radiation dose (EUD) and changes in cognitive scores in patients treated for PFTs. In particular, we highlighted a link between working memory decline and radiation dose in the orbitofrontal region, whereas the decline in processing speed seemed more related to irradiation of the temporal lobes and the PF.

### Effect of Clinical Variables on Cognitive Score Changes

Consistently with previous studies (5, 6), the FSIQ decline depended on the delay between the two IQ tests. As shown in previous studies (5, 38), chemotherapy does not seem to have a significant negative impact on PSI and WMI functioning. The surprising positive effect of chemotherapy on FSIQ change might be linked to the positive impact of repeated measurements, also known as the carry over effect (or IQ test–retest) (39). Children acquired expertise concerning neuropsychological task along many neuropsychological tests, improving their performances. Therefore, the change in cognitive scores of each patient calculated from the difference between the first and last scores was positive. A large portion of children with chemotherapy alone showed an IQ improvement which confirms the absence of cognitive effect of chemotherapy (5, 38).

This also explains the unexpected negative effect of age at diagnosis on FSIQ change, as children treated with chemotherapy alone are usually young (below 5 years) at diagnosis.

### ROIs EUD and Cognitive Score Changes

All components seem to have specific impacts on changes of the working memory score (WMI). The radiation distribution pattern involving the left orbitofrontal regions (*PC3-_EUD_*) had the most negative impact on working memory. Interestingly, this result could be in line with Mabbott et al.?’s findings (40). They observed that working memory performance over time was different according to the tumor location in children treated for a central nervous system germ cell tumor. Patients with pineal tumors showed early, but stable, working memory deficit, whereas patients with suprasellar tumors experienced a significant working memory decline over time. Mabbott et al. suggested the observed decline was related to the radiation field rather than to the tumor location (40). In addition, this observation fits well with the compelling neuroimaging evidence of orbitofrontal implication in tasks relying on working memory [for meta-analysis, see Ref. (41, 42)]. *PC1-_EUD_*, however, corresponds to a distributed radiation pattern across the whole brain, suggesting that a global increase of radiation dose (EUD) impacts working memory negatively. From its patterns of spatial radiation distribution, this last component could be interpreted as CSI dose variability across subjects. However, such an overall radiation effect does not allow us to distinguish specifically irradiated brain networks that could be particularly involved in working memory impairment.

More specific brain network radiation patterns are found to influence processing speed. The large impact of *PC3-_EUD_* is strongly related to radiation to the temporal lobes and the PF. Previous studies have shown significant associations between radiation dose to the temporal lobe and processing speed impairments (16, 17, 43). The cerebellum has also been shown to play a role in processing speed capabilities (44). Importantly, temporal lobe regions are close to the PF upon which the dose was escalated. Thus, *PC3-_EUD_* impact could also reflect the radiation field boost trajectory to the PF across subjects. This would support the hypothesis that the volume receiving the highest dose has the greatest impact on cognitive functions. Accordingly, these findings would support current volume–reduction efforts.

Finally, *PC2-_EUD_* and *PC3-_EUD_* carry the exact same negative effect on IQ change. As reported earlier, *PC2-_EUD_* that includes the temporal lobes and the PF showed the most significant impact on processing speed changes. As for processing speed, previous studies have found associations between radiation dose to the temporal lobe and PF and IQ impairments (16, 18). In the same way, the role on IQ impairment of *PC3-_EUD_*, which strongly involves the left orbitofrontal cortex, is somehow expected, as many VBM studies in adults and adolescents have shown a link between IQ and gray matter density in this region (45–47). Alternatively, the equal contribution of these two components on ΔFSIQ might be the expression of an averaging effect as FSIQ is a composite index encompassing both working memory and processing speed.

Higher EUD in the superior occipital and parietal regions did not seem to be associated with lower cognitive scores. We may note that Armstrong et al. (17) did not find any significant association between occipito-parietal radiation dose and cognitive or social problems either. In addition, with the same amount of radiation dose, the parietal lobe white matter was shown to be less affected compared to frontal lobe in medulloblastoma (48, 49). Thus, it would be interesting to test whether the parietal lobe is less susceptible to radiation than other regions.

There are limitations in this study, and results should be interpreted with caution. First, the small size and heterogeneity of the patient population make it difficult to control for other variables that could affect the scores (i.e., hydrocephalus shunt, education, rehabilitation, surgical approach, molecular group, etc.). Moreover, considering only PFT patients prevented us from taking into account several potentially confounding variables such as type and localization of the tumor. However, this was a disadvantage regarding the large spatial correlations between close irradiated regions induced by similar radiation protocol. We could not access separately specific regions that are known to play an important role in working memory [e.g., dorsolateral area (50)] or processing speed [e.g., left middle frontal gyrus (51)]. Second, noise could be induced by intersubject variability of the brain morphology, even if we minimized possible segmentation errors by using atlases specific to age groups. Finally, we have to acknowledge that seven patients (that received hyperfractionated radiotherapy, HFRT) had the same total dose and could be considered as a subgroup that could influence the results (see Figure S1 in Supplementary Material). We recognize the possibility that the HFRT subgroup smaller variance might influence the result in other less crucial ways (see Figure S2 in Supplementary Material).

## Conclusion

This study confirms two cases for which there is a relationship between the radiation dose in particular brain areas and specific cognitive decline. The first case shows a correlation between orbitofrontal radiation and working memory decline, whereas the second case portrays a correlation between temporal lobe and PF radiation and slower processing speed. As this study is exploratory, it does aim to provide information regarding brain regions to avoid, but to describe relationships between radiation and cognitive function. The relationship between the cognitive profiles and the irradiation of these regions should be further confirmed in a prospective randomized with both, a bigger cohort and different radiation protocols.

## Ethics Statement

This retrospective study was approved by the Comite de Protection des Personnes CPP no. 14973 (Ile de France, France). All patient’s parents gave a written informed consent in accordance with the Declaration of Helsinki.

## Author Contributions

All authors carried a substantial contribution to the article and approved the final version of the manuscript. None of them has any conflict of interest. Guarantor of integrity of entire study: EDS, MN, and LH-P. Study concepts and design or data collection or data analysis and interpretation: all authors. Data preprocessing: EDS, CR, AG, and CP. Statistical analysis: EDS and MP-G. Drafting the work or revising it critically for important intellectual content: all authors.

## Conflict of Interest Statement

The authors declare that the research was conducted in the absence of any commercial or financial relationships that could be construed as a potential conflict of interest. The reviewer, JB, and the handling editor declared their shared affiliation, and the handling editor states that the process nevertheless met the standards of a fair and objective review.
