# Supplementary material for: Relationships between Regional Radiation Doses and Cognitive Decline in Children Treated with Cranio-Spinal Irradiation for Posterior Fossa Tumors
Source: Front Oncol. 2017 Aug 18;7:166. doi: 10.3389/fonc.2017.00166 (PMC5563322; doi:10.3389/fonc.2017.00166)

Supplementary data

Table S1. Design of common anatomical references extracted from the PCA. Abbreviation: L=left, R=right, PC=Principal Component, EUD = Equivalent Uniform Dose

EUD - PCs	**Correlation sign**	Regions **strongly** correlated with PC	**Common anatomical reference**
PC1	Positive	All regions	
PC2	Positive	RL fusiform gyrus, RL parahippocampal gyrus, RL lingual gyri, R hippocampus, R middle temporal gyrus, L hippocampus, R inferior occipital gyrus, L middle temporal gyrus, L inferior occipital gyrus, L inferior temporal gyrus,	Inferior occipital and temporal regions
	Positive	Brainstem, cerebellum	Posterior fossa
	Negative	L superior occipital gyrus, L supramarginal gyrus, L angular gyrus	Left superior occipital and inferior parietal regions
PC**3**	Negative	RL precuneus	Mesial parietal region
	Positive	RL gyrus rectus, L middle orbitofrontal	Orbitofrontal cortex

Table S2. Correlations between PCs (n = 3) and EUD in each ROI (n = 57).

Regions	EUD		
	PC1-$_{EUD}$	PC2-$_{EUD}$	PC3-$_{EUD}$
**Brainstem**	0,58	0,78	0,16
**Cerebellum**	0,61	0,69	0,25
**L angular gyrus**	0,82	-0,35	-0,32
**L caudate**	0,90	-0,30	0,16
**L cingulate gyrus**	0,93	-0,10	-0,26
**L cuneus**	0,85	-0,16	-0,38
**L fusiform gyrus**	0,57	0,80	0,11
**L gyrus rectus**	0,83	-0,22	0,45
**L hippocampus**	0,85	0,47	0,11
**L inferior frontal gyrus**	0,90	-0,35	0,11
**L inferior occipital gyrus**	0,67	0,66	0,09
**L inferior temporal gyrus**	0,70	0,55	0,32
**L insular cortex**	0,94	-0,01	-0,12
**L lateral orbitofrontal gyrus**	0,86	-0,18	0,30
**L lingual gyrus**	0,64	0,67	-0,24
**L middle frontal gyrus**	0,90	-0,36	0,16
**L middle occipital gyrus**	0,86	-0,14	-0,20
**L middle orbitofrontal gyrus**	0,85	-0,26	0,41
**L middle temporal gyrus**	0,78	0,55	0,12
**L parahippocampal gyrus**	0,60	0,78	0,06
**L postcentral gyrus**	0,83	-0,53	0,04
**L precentral gyrus**	0,84	-0,51	0,05
**L precuneus**	0,82	-0,04	-0,52
**L putamen**	0,95	0,02	0,02
**L superior frontal gyrus**	0,91	-0,32	0,18
**L superior occipital gyrus**	0,76	-0,59	-0,14
**L superior parietal gyrus**	0,84	-0,48	-0,09
**L superior temporal gyrus**	0,87	0,14	-0,18
**L supramarginal gyrus**	0,73	-0,57	-0,24
**R angular gyrus**	0,92	-0,01	-0,29
**R caudate**	0,89	-0,32	0,18
**R cingulate gyrus**	0,94	-0,04	-0,27
**R cuneus**	0,77	0,10	-0,51
**R fusiform gyrus**	0,53	0,83	0,06
**R gyrus rectus**	0,84	-0,22	0,44
**R hippocampus**	0,77	0,52	0,03
**R inferior frontal gyrus**	0,93	-0,25	0,11
**R inferior occipital gyrus**	0,61	0,74	0,11
**R inferior temporal gyrus**	0,79	0,55	0,21
**R insular cortex**	0,96	-0,03	-0,08
**R lateral orbitofrontal gyrus**	0,93	-0,23	0,15
**R lingual gyrus**	0,58	0,76	-0,14
**R middle frontal gyrus**	0,91	-0,34	0,16
**R middle occipital gyrus**	0,89	0,21	-0,20
**R middle orbitofrontal gyrus**	0,89	-0,31	0,31
**R middle temporal gyrus**	0,78	0,56	0,03
**R parahippocampal gyrus**	0,60	0,77	0,03
**R postcentral gyrus**	0,93	-0,32	0,11
**R precentral gyrus**	0,92	-0,32	0,12
**R precuneus**	0,85	-0,01	-0,45
**R putamen**	0,95	-0,04	0,03
**R superior frontal gyrus**	0,91	-0,33	0,19
**R superior occipital gyrus**	0,92	-0,18	-0,15
**R superior parietal gyrus**	0,95	-0,24	-0,03
**R superior temporal gyrus**	0,86	0,32	-0,21
**R supramarginal gyrus**	0,93	0.18	0.14
**Supplementary label**	0.17	0,31	-0,30

Figure S1. Total 2Gy equivalent biological dose (PF + CSI) for each patient.

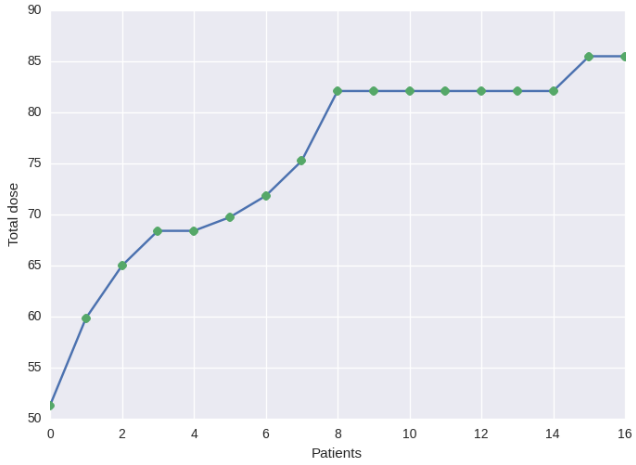

Figure S2. **Patients coordinates projected to the principal component space and colored according to the type of fractionation (CF vs HFRT) in order to evaluate if the reported first three PCA components were driven by the HFRT subgroup.** The HFRT and CF distributions have similar mean values in the components with an expected difference in variance. Based on this we would not expect any of the main patterns of radiation captured by the first three PCA components to be importantly driven by a group difference. If it was the case we would expect to find a bimodal distribution. Nonetheless, we recognize the possibility that the HFRT subgroup smaller variance might influence the result in other less crucial ways

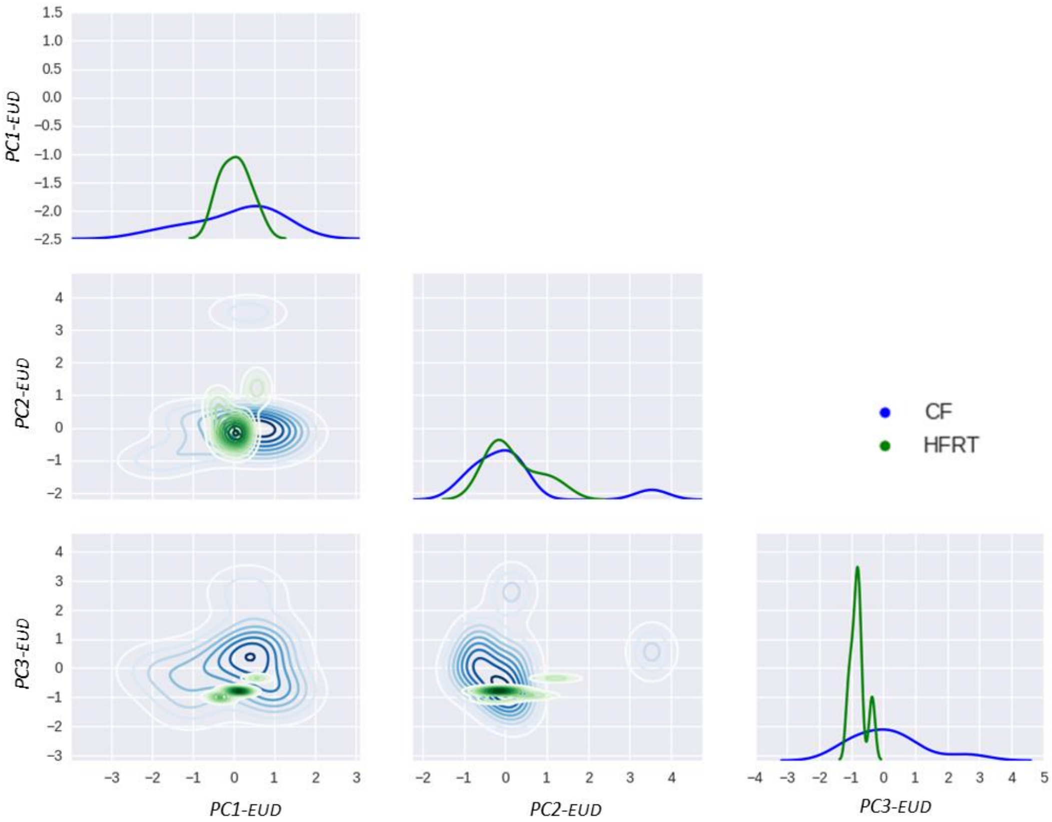

Supplement: Supplementary file 1 [file Data_Sheet_1.PDF]
